# Self-reported anal cancer screening experiences in MSM: recency, follow-up, and methodological insights

**DOI:** 10.3389/fonc.2026.1689760

**Published:** 2026-04-15

**Authors:** Sarah L. Bennis, M. Kumi Smith, Xianghua Luo, Jason V. Baker, Eva A. Enns, Shalini Kulasingam

**Affiliations:** 1 Division of Epidemiology and Community Health, School of Public Health, University of Minnesota, Minneapolis, MN, United States; 2 Division of Biostatistics and Health Data Science, School of Public Health, University of Minnesota, Minneapolis, MN, United States; 3 Masonic Cancer Center, University of Minnesota, Minneapolis, MN, United States; 4 Division of Infectious Diseases, Hennepin Healthcare, Minneapolis, MN, United States; 5 Division of Health Policy and Management, School of Public Health, University of Minnesota, Minneapolis, MN, United States; 6 Department of Tropical Medicine and Infectious Diseases, Celia Scott Weatherhead School of Public Health and Tropical Medicine, Tulane University, New Orleans, LA, United States

**Keywords:** anus neoplasms, early detection of cancer, health inequities, HIV, human papillomavirus viruses, sexual and gender minorities

## Abstract

**Introduction:**

Men who have sex with men (MSM) are disproportionately affected by human papillomavirus (HPV)-associated anal cancer. Prior to national anal cancer screening guidelines, literature suggested that the overall utilization of screening was low, with slightly higher uptake in MSM living with human immunodeficiency virus (HIV). The objective of this study was to report the recency of anal cancer screening by various screening methods and describe differences in self-reported screening histories by HIV status in MSM. We also aimed to comment on the use of cross-sectional methods for research on anal cancer screening utilization in MSM.

**Methods:**

This cross-sectional survey recruited MSM in Minnesota from two online dating sites to participate in a single survey on anal cancer screening experiences. Fisher’s exact tests and t-tests were used to examine differences in demographics and screening experiences by HIV status.

**Results:**

A total of 52 MSM participated in the survey, and 38 provided data on previous anal cancer screening experiences. Most participants were white (65.4%), gay (88.5%), and HIV-negative (69.8%). Approximately 57% of MSM living with HIV self-reported an experience with anal cancer screening in the last year compared to 37.5% of MSM living without HIV (p = 0.32). Screening was often recommended by HIV providers for MSM living with HIV (66.7%) versus primary care providers for HIV-negative MSM (47.2%, p < 0.01). MSM living with HIV more often reported a history of an abnormal result compared to HIV-negative MSM (71.4% *vs*. 29.2%, p = 0.018).

**Discussion:**

Our findings suggest that MSM in Minnesota are utilizing anal cancer screenings in accordance with current recommendations. MSM living with HIV were more likely to report a history of HPV-related infections and abnormal results, supporting continued prioritization of screening in this population. The differences in recommending providers by HIV status highlight the need for additional training to support equitable and consistent practices. Finally, we suggest the use of alternative methods to document future anal cancer screening utilization in MSM.

## 1 Introduction

Human papillomavirus (HPV), a sexually transmitted virus, causes up to 91% of anal cancers (1, 2). Anal infections with high-risk HPV (hrHPV) are more prevalent in men who have sex with men (MSM) compared to men who have sex with women (MSW), with prevalence estimated at 6.9% in MSW, 41.2% in MSM living without human immunodeficiency virus (HIV), and 74.3% in MSM living with HIV (3). Compared to MSW, MSM experience greater burden of anal hrHPV infections due to several factors including higher prevalence of HIV (98 times higher for MSM *vs*. MSW), which impedes immune response to infections (4, 5); a greater numbers of lifetime sex partners that increase exposure to the virus (median 45 for MSM *vs*. 8 for MSW) (6, 7); and low uptake of HPV vaccinations in men generally (27% of men ages 18–26 received ≥1 dose in 2018) (8, 9). While most HPV infections clear spontaneously, a small proportion can persist and eventually develop into anal precancers or cancer. The estimated incidence rates are 85 cases/100,000 for MSM living with HIV per year and 12 cases/100,000 for MSM living without HIV per year (10, 11). With detection through screening, anal precancerous lesions are treatable and can prevent progression to invasive cancer (12). The first anal cancer screening guidelines focusing on key populations, like MSM, were published in 2024 (13). With the introduction of guidelines, there are new opportunities to explore methods for tracking anal cancer screening uptake and MSM’s experiences with screening more broadly.

The Centers for Disease Control and Prevention (CDC) and the International Anal Neoplasia Society (IANS) independently published the first routine recommendations for anal cancer screening. The CDC and IANS recommend anal cancer screening for people living with HIV, including MSM, beginning at age 35 years with cytology or co-testing with both cytology and HPV testing (13, 14). The recommendations also include screening high-risk groups living with HIV (CDC) and without HIV (IANS) beginning at age 45 (13, 14). Although the IANS published that high-risk HPV testing alone is acceptable, the CDC notes that HPV testing alone should not be conducted, nor should any screening take place without access to follow-up high-resolution anoscopy (HRA) (13, 14). Guidelines may begin to standardize the age to start screening, what methods to use, and the time between screenings; however, recent studies have not assessed these metrics.

Estimates of screening utilization in MSM in the literature are highly variable. In a survey of MSM (with and without HIV) from 2009, only 14% of men reported ever receiving an anal Pap test (15). In 2018, upwards of 52% of MSM living with HIV at a specialized care clinic reported an experience with anal cancer screening in the past year (16). Several additional studies have reported inconsistent estimates on the proportion of men ever screened for anal cancer or screened in the past year, with high variability in estimates based on regional location (16–18). Inconsistent screening prevalence reports potentially reflect the lack of standardized screening guidelines prior to 2024, which may also vary by HIV status (19). Access to anal cancer screening and HRA in Minnesota is not well documented. A study conducted at community-based events locally indicated that <20% of MSM who reside in Minnesota and engage in anal intercourse had been screened in mid-2024 (20). Currently, screening and follow-up services are largely centered in urban areas for MSM living with HIV, such as “Save the Bottoms” at the University of Minnesota and the Positive Care Center at Hennepin Healthcare (21, 22). Since the publication of guidelines, few studies have re-examined the recency of screening.

The objective of this cross-sectional study was to report the recency of anal cancer screening (<1 year ago *vs*. ≥1 year ago) by various methods and describe differences by HIV status in MSM in Minnesota. We also had the opportunity to ask MSM about the provider who recommended screening and abnormal screening result follow-up. Lastly, we aimed to comment on the utility of survey methods and self-reported anal cancer screening outcomes for future research. This study will provide updated estimates of self-reported screening recency and follow-up care to inform local policies and strategies to improve anal cancer screening experiences in MSM.

## 2 Methods

MSM in Minnesota were recruited to participate in this cross-sectional study through two online dating sites, Scruff and Jack’d (Perry Street Software, New York City, NY, USA). The dating sites used a geotargeted algorithm to display three recruitment ads (Appendix A) to all active users during the 33 days of recruitment across 3 months (December 2024, March 2025, and April 2025). The eligibility screener and survey were programmed in Qualtrics (Provo, UT, USA). Individuals meeting all inclusion criteria were eligible to participate: 1) current residence in Minnesota, 2) aged 18 years or older, 3) sex assigned at birth is male, 4) reported a history of sex with a man, 5) self-reported a history of anal cancer screening, 6) knew their HIV status (HIV+ or HIV−), and (7) passed survey CAPTCHA validation. On average, it took participants 10 minutes to complete the survey. Participants were compensated up to $20 for their contribution to the study. Prior to recruitment, the University of Minnesota Institutional Review Board and the Cancer Protocol Review Committee (STUDY00023836) approved all study materials.

### 2.1 Survey items

The survey collected data on demographics, self-reported sexual behaviors, sexual health history, and HPV vaccination history (**Table 1**). Previously published questions from national surveys like the National Health and Nutrition Examination Survey (23), the Behavioral Risk Factor Surveillance System (24), and the National Survey of Sexual Attitudes and Lifestyles (25) were utilized to ensure accurate ascertainment of data on demographics, sexual behaviors, and HPV vaccinations. Validated questions on anal cancer screening experiences are lacking in the literature; therefore, we consulted experts in the field (colorectal surgeon and infectious disease provider) to review survey questions and response options for accuracy.

**Table 1:** Demographic characteristics of MSM in cross-sectional study (N = 52).

Characteristic	Overall		HIV-positive (n = 16)		HIV-negative (n = 36)		p-Value
	N	%	N	%	N	%	
Current age (median, IQR)							0.27
	45.5 (39.5–53)		44.5 (41–60.5)		46 (38–52.5)		
Race							
American Indian or Alaska Native	1	1.9%	1	6.3%	0	0.0%	0.68
Asian	3	5.8%	0	0.0%	3	8.3%	
Black or African American	6	11.5%	2	12.5%	4	11.1%	
Hispanic/Latino	3	5.8%	1	6.3%	2	5.6%	
More than one	5	9.6%	1	6.3%	4	11.1%	
White	34	65.4%	11	68.8%	23	63.9%	
Sexual orientation							
Gay	46	88.5%	16	100.0%	30	83.3%	0.61
Bisexual	2	3.8%	0	0.0%	2	5.6%	
Pansexual	1	1.9%	0	0.0%	1	2.8%	
Queer	3	5.8%	0	0.0%	3	8.3%	
Gender identity							
Cisgender man	45	90.0%	13	81.3%	32	88.9%	0.62
Non-binary	1	2.0%	0	0.0%	1	2.8%	
Two-spirit	1	2.0%	1	6.3%	0	0.0%	
Other identity	3	6.0%	1	6.3%	2	5.6%	
Education							
High school or GED	6	11.5%	2	12.5%	4	11.1%	0.03*
Some college	5	9.6%	3	18.8%	2	5.6%	
Technical degree	1	1.9%	1	6.3%	0	0.0%	
Associate’s degree	3	5.8%	1	6.3%	2	5.6%	
Bachelor’s degree	20	38.5%	8	50.0%	12	33.3%	
Graduate or professional degree	17	32.7%	1	6.3%	16	44.4%	
History of smoking							
Yes	22	42.3%	10	62.5%	12	33.3%	0.07
No	30	57.7%	6	37.5%	24	66.7%	
HIV status							
HIV-positive	16	30.8%	–		–		–
HIV-negative	36	69.2%	–		–		
Taking ART/HAART (only HIV+)							
Yes	–		15	93.8%	–		–
No	–		1	6.3%	–		
Taking PrEP (only HIV−)							
Yes	–		–		26	74.3%	–
No	–		–		9	25.7%	
History of STIs (excluding HPV and HIV)							
Yes	44	84.6%	15	93.8%	29	80.6%	0.41
No	8	15.4%	1	6.2%	7	19.4%	
History of HPV infection or related disease							
Yes	17	32.7%	10	62.5%	7	19.4%	<0.01*
No	34	65.4%	5	31.3%	29	80.6%	
Not sure/don’t remember	1	1.9%	1	6.3%	0	0.0%	
Health insurance							
Insured	50	96.2%	16	100.0%	34	94.4%	>0.99
Uninsured	2	3.8%	0	0.0%	2	5.6%	
Self-reported HPV vaccination status							
Vaccinated	25	48.1%	7	43.8%	18	50.0%	0.17
Unvaccinated	23	44.2%	6	37.5%	17	47.2%	
Unsure	4	7.7%	3	18.8%	1	2.8%	
Self-reported number of doses (only vaccinated)							
1	2	8.0%	0	0.0%	2	5.6%	0.60
2	7	28.0%	1	6.3%	6	16.7%	
3+	14	56.0%	5	31.3%	9	25.0%	
Not sure/don’t remember	2	8.0%	1	6.3%	1	2.8%	
Self-reported age at first HPV dose (median, IQR)							
	32 (25–40)		35 (30–38)		31 (22–40)		0.25
Age at sexual debut (median, IQR)							
	17 (14–19.5)		16 (12–18.5)		17 (14.5–21)		0.51
Lifetime number of anal sex partners							
2–5	3	5.8%	1	6.3%	2	5.6%	0.08
6–10	0	0.0%	0	0.0%	0	0.0%	
11–25	6	11.5%	1	6.3%	5	13.9%	
26–50	5	9.6%	1	6.3%	4	11.1%	
51–100	9	17.3%	0	0.0%	9	25.0%	
>100	29	55.8%	13	81.3%	16	44.4%	
Number of RAI partners in last year							
0	10	19.2%	3	18.8%	7	19.4%	0.57
1	5	9.6%	2	12.5%	3	8.3%	
2–5	14	26.9%	3	18.8%	11	30.6%	
6–10	7	13.5%	2	12.5%	5	13.9%	
11–25	8	15.4%	2	12.5%	6	16.7%	
26–50	6	11.5%	4	25.0%	2	5.6%	
51–100	0	0.0%	0	0.0%	0	0.0%	
>100	2	3.8%	0	0.0%	2	5.6%	
Condom use in last year (only those with ≥1 RAI partner in past year)							
Always	2	4.8%	0	0.0%	2	5.6%	0.38
Sometimes	17	40.5%	4	25.0%	13	36.1%	
Never	23	54.8%	9	56.3%	14	38.9%	

IQR, interquartile range; GED, General Educational Development; HIV, human immunodeficiency virus; STI, sexually transmitted infection; HPV, human papillomavirus; RAI, receptive anal intercourse; PrEP, pre-exposure prophylaxis; ART/HAART, antiretroviral therapy or highly active antiretroviral therapy.

*p-Value < 0.05.

#### 2.1.1 Deduplication and validation

Survey responses were evaluated for uniqueness and validity prior to data cleaning. Using RelevantID technology provided by Qualtrics (highlighting bots or duplicates) (26), time to complete the survey, IP address, and email addresses, we assigned points to each metric to create a continuous scale indicating whether surveys were bots, duplicates, or unique.

### 2.2 Statistical analysis

We summarized participant characteristics overall and by HIV status (HIV+ or HIV−) (**Table 1**). Due to the small sample sizes, we utilized unadjusted Fisher’s exact tests and t-tests to examine the associations between HIV status, demographics, and anal cancer screening outcomes for participants with complete data. We also compared the demographics of participants with and without missing anal cancer outcomes using Fisher’s exact tests and t-tests (**Supplementary Table 1**). All tests were two-sided, and p-values < 0.05 were considered statistically significant.

## 3 Results

A total of 475 individuals initiated the screener (452 determined to be unique responses), with 418 completing all eligibility determination questions. Of those who completed the eligibility screener, 104 were deemed eligible, and 56 proceeded to consent to participate. In total, 52 participants proceeded to the main survey. After reviewing survey responses, 38 participants provided information on anal cancer screening experiences (n = 1 did not answer questions, n = 2 removed because they stated they had a colonoscopy, and n = 11 stated they had not been screened for anal cancer after reviewing descriptions of screening methods).

### 3.1 Demographics

Participants in the study were largely white (65.4%), gay (88.5%), and cisgender men (90.0%) with college degrees (71.2%) and health insurance (96.2%) (**Table 1**). The median age of participants was 45.5 years. Approximately 1/3 of the sample self-reported living with HIV (30.8%), and demographic characteristics were similar across MSM living with and without HIV.

There were no statistically significant differences in smoking history between MSM living with and without HIV (62.5% *vs*. 33.3%, p-value = 0.07). At the time of survey completion, 93.8% of the MSM living with HIV reported taking antiretroviral therapy (ART). For MSM living without HIV, 74.3% reported taking pre-exposure prophylaxis (PrEP).

The sample reported high levels of sexual activity. The median age at sexual debut was 17 years. More than 55% of men reported >100 lifetime anal sex partners, and 71.2% reported two or more partners with whom they had had receptive anal intercourse in the past year. Very few men reported consistently using condoms in the last year (4.8%).

### 3.2 HPV vaccinations

A majority of men self-reported their HPV vaccination status, with a small minority indicating uncertainty about their vaccination history (**Table 1**). Overall, 48.1% of men self-reported receipt of at least one dose of the HPV vaccine. The proportion of vaccinated and unvaccinated men was similar across HIV status. Among vaccinated MSM, the median age at first HPV dose was 32 years (IQR 25-40 years).

### 3.3 Anal cancer screening

Anal cancer screening was typically initiated by MSM in their late 30s, and the median number of screenings in their life was 2.5 (**Table 2**). For MSM living with HIV, it was typically first recommended by an infectious disease or HIV provider (92.9%), whereas MSM living without HIV reported a primary care provider (66.7% first recommended screening (p-value < 0.01).

**Table 2:** Self-reported anal cancer screening experiences in cross-sectional sample of MSM (N = 38).

Characteristic	Overall		HIV-positive (n = 14)		HIV-negative (n = 24)		p-Value
	N	%	N	%	N	%	
Who first recommended screening?							
Infectious disease/HIV provider	17	47.2%	13	92.9%	4	16.7%	<0.01*
Primary care provider	17	47.2%	1	7.1%	16	66.7%	
Family member	1	2.8%	0	0.0%	1	4.2%	
Don’t remember	1	2.8%	0	0.0%	1	4.2%	
Age at first screening (median, IQR)							
	38 (30–45)		38 (32–46)		37 (29.5–45)		0.31
Time since most recent anal cancer screening by any method (including HRA)							
<1 year	17	44.7%	8	57.1%	9	37.5%	0.32
≥1 year	21	55.3%	6	42.9%	15	62.5%	
Time since most recent anal cancer screening with Pap							
<1 year	13	50.0%	6	66.7%	7	41.2%	0.62
≥1 year	12	46.1%	3	33.3%	9	52.9%	
Don’t remember	1	3.9%	0	0.0%	1	5.9%	
Time since most recent anal cancer screening with HPV test							
<1 year	4	30.8%	2	50.0%	2	22.2%	0.53
≥1 year	9	69.2%	2	50.0%	7	77.8%	
Time since most recent anal cancer screening with DARE							
<1 year	3	23.1%	1	50.0%	2	18.2%	0.54
≥1 year	9	69.2%	1	50.0%	8	72.7%	
Don’t remember	1	7.7%	0	0.0%	1	9.1%	
Time since most recent HRA							
<1 year	7	63.6%	2	33.3%	5	100.0%	0.06
≥1 year	4	36.4%	4	66.7%	0	0.0%	
Lifetime number of anal cancer screens (median, IQR)							
	2.5 (1–5)		2.5 (1–5)		2.5 (1–5)		0.41
Ever screened with Pap?							
Yes	26	68.4%	9	64.3%	17	70.8%	0.73
No	12	31.6%	5	35.7%	7	29.2%	
Ever screened with HPV test?							
Yes	13	34.2%	4	28.6%	9	37.5%	0.73
No	25	65.8%	10	71.4%	15	62.5%	
Ever screened with DARE?							
Yes	13	34.2%	2	14.3%	11	45.8%	0.27
No	25	65.8%	12	85.7%	13	54.2%	
Ever undergone HRA?							
Yes	11	28.9%	6	42.9%	5	20.8%	0.15
No	27	71.1%	8	57.1%	19	79.2%	
History of abnormal screening result							
Yes	17	44.7%	10	71.4%	7	29.2%	0.02*
No	21	55.3%	4	28.6%	17	70.8%	
Abnormal screening follow-up							
Rescreen immediately	3	17.6%	3	30.0%	0	0.0%	0.13
HRA and rescreen immediately	1	5.9%	0	0.0%	1	14.3%	
Rescreen in 1 year	2	11.8%	2	20.0%	0	0.0%	
HRA and rescreen in 1 year	4	23.5%	1	10.0%	3	42.9%	
HRA only	6	35.3%	4	40.0%	2	28.5%	
No follow-up	1	5.9%	0	0.0%	1	14.3%	
History of anal cancer							
Yes	2	5.3%	1	7.1%	1	4.2%	1
No	36	94.7%	13	92.9%	23	95.8%	

IQR, interquartile range; HIV, human immunodeficiency virus; HPV, human papillomavirus; DARE, digital anorectal examination; HRA, high-resolution anoscopy; Pap, Papanicolaou test.

* p-Value < 0.05.

MSM living with and without HIV reported initiating anal cancer screening at approximately the same age and had the same total number of screens in their lifetime (both p-values > 0.05; **Table 2**). More MSM reported their last screening was one or more years ago (55.3%), with a higher proportion of MSM living with HIV being screened less than 1 year ago (57.1% *vs*. 37.5%). MSM frequently reported a history of screening with anal Pap testing (68.4%), and less frequently with HPV testing (34.2%) or digital anorectal examination (DARE) (34.2%). There were no statistically significant differences in past-year screening by any method. Notably, a greater proportion of MSM living with HIV self-reported an abnormal screening result compared to MSM without HIV (71.4% *vs*. 29.2%, p-value = 0.018). Following an abnormal result, men frequently reported referral to HRA (35.3%), rescreening (29.4%), or a combination of HRA and rescreening (29.4%). While there were no statistically significant differences, the proportion of men who reported any history of HRA was slightly higher in HIV-positive MSM (42.9%) compared to HIV-negative MSM (20.8%). In the last year, though, most MSM who underwent HRA were HIV-negative.

## 4 Discussion

The primary aim of this cross-sectional study was to report the recency of anal cancer screening and follow-up steps for abnormal results from the perspectives of MSM living with and without HIV in Minnesota. Overall, a large proportion of MSM reported an experience with anal cancer screening in the past year. We noted no significant differences in the self-reported time since last screening, methods used for screening, or the lifetime number of screenings between MSM living with and without HIV. We found that MSM living with HIV more frequently reported a history of abnormal screening results compared to MSM living without HIV. While MSM living with HIV were more often referred for HRA, the difference was not significant. However, there were notable differences in the providers who first recommended anal cancer screening, with MSM living with HIV commonly reporting an infectious disease or HIV provider as the recommender, whereas HIV-negative MSM most frequently reported a primary care provider recommended screening. Finally, given the challenges of recruiting a convenience sample using survey methods, we suggest alternative methods to continue documenting changes in anal cancer screening utilization.

Data from our study provide insight into the recency of anal cancer screenings in MSM since the release of new screening guidelines in 2024. In the 2010s, studies have reported that while acceptance of anal cancer screening was high (83%–85%), experiences with ever being screened ranged from 10% to 15% for MSM living without HIV and 30% to 39% for MSM living with HIV (15, 19). Later in the decade, one study from a specialized HIV clinic reported that 51% of MSM had a history of anal Pap screening; however, these data likely reflected the specialized data source that targeted high-risk men and did not report estimates for MSM without HIV (16). Conversely, using CDC Medical Monitoring Project data from 2019, Rim and colleagues reported that only 7.7% of U.S. MSM aged 35 or older living with HIV were screened for anal cancer with a Pap test within the past year (17). Moving into the early 2020s, a secondary analysis of survey data from sexual and gender minority men from Pennsylvania suggested more modest screening utilization, with 10.02% of HIV-negative men and 33.63% of men living with HIV reporting an anal cancer screening within the last year (18). In the present study, we observed more frequent screening in the past year in MSM living with HIV compared to without HIV (57.1% *vs*. 37.5%) and noted substantial increases compared to previous estimates in the literature. While it is possible that MSM reported experiences with screening prior to publication of standardized guidelines, a large proportion of MSM were screened within the last year, highlighting the need for additional research to continue tracking patterns of screening recency. Additionally, our findings provide context for geographical variations in screening recency, underscoring the value of localized estimates to inform targeted screening programs and ensure adequate follow-up for MSM.

While we observed overall increases in screening compared to previous literature, our results suggest that MSM living with and without HIV have similar experiences with screenings. In the present study, MSM living with and without HIV initiated screening at the same age and reported the same number of lifetime screens, and there were no statistically significant differences in recency of screening overall or by any specific method. While this may reflect the small sample size that was underpowered to detect differences, there may be other factors supporting the similarities. The participants in this study were highly educated, had health insurance, and engaged in care, which may lead to similar healthcare-seeking behaviors. Education and consistent access to care have both been previously documented as facilitators to screening (18, 27); in one qualitative study, an HIV-negative man stated that “[he] know[s] that no matter how it [HRA results] comes back [ … ] [he has] the resources to deal with it” within the context of healthcare support and flexibility to take time from work for follow-up appointments (28). Additionally, LGBTQ-focused healthcare providers are often concentrated in urban areas, and the Minneapolis/St. Paul area is no exception (29). As a result, MSM with and without HIV may obtain care from providers with similar anal cancer screening practices. While we did not observe differences in the proportion of men screened within the past year compared to more than 1 year ago, efforts to track changes in screening should continue, as it may begin to increase in accordance with current guidelines.

Importantly, we did observe a greater proportion of MSM living with HIV reporting a history of HPV infection or related disease and a history of abnormal screening results compared to HIV negative MSM, which is consistent with existing literature. In a cohort study of MSM, 21% of HIV-negative MSM had abnormal anal cytology results compared to 36% of HIV-positive MSM (30). MSM living with HIV have a higher prevalence of HPV infections overall (55%–74.3%) and infection with oncogenic HPV-16 (28.5%–35.4%) compared to MSM living without HIV (HPV overall = 41.2%–50%; HPV-16 = 12.5%–13.7%) (3, 4, 6, 31, 32). Due to slowed immune response in people living with HIV, clearance of oncogenic HPV may take longer or remain persistent, leading to anal dysplasia (4, 31). In a meta-analysis (2021), the pooled prevalence of anal high-grade intraepithelial lesion (HSIL) was 22.4% in MSM living with HIV and 11.3% in HIV-negative MSM (3). Typically, abnormal results would be referred to HRA for diagnosis and treatment, but the lack of trained providers is a major barrier to implementing more robust screening programs. In a nationally representative sample of people living with HIV, nearly one-third of those screened for anal cancer did not have access to follow-up HRA (17). In our study, HIV-negative MSM reported the majority of HRA use in the past year. Future directions for research may consider documenting the availability of HRA and exploring utilization by HIV status to ensure that access to follow-up care for those at greatest risk of anal dysplasia and cancer is available.

Larger studies are needed to confirm our findings on MSM’s experiences with anal cancer screenings, and efforts to support consistent screening for MSM most at risk should be considered. Most MSM find anal cancer screening acceptable, but lack of anal cancer knowledge, fear of abnormal results, and concerns around painful procedures are often cited as barriers to screening (15, 17, 27). As screening utilization potentially increases in the future, the importance of provider communication to support patients should not be overlooked. A majority of MSM living with HIV in our study reported that an infectious disease provider first recommended anal cancer screening, compared to more primary care provider recommendations reported by HIV-negative MSM. Provider recommendations are associated with anal cancer screening uptake, but may not be sufficient to support consistent annual screening (33, 34) or accurate recall of screening history. In the present study, MSM expressed confusion about anal cancer screenings, occasionally conflating anal cancer with colorectal cancer screening. While the literature is lacking research on provider communication approaches to promote anal cancer screening and the quality of these discussions, adherence has been shown to improve when providers thoroughly explain the procedures (33). Education to providers on how to accurately describe anal cancer screenings may quell concerns over anal cancer screening that may otherwise pose barriers.

We also suggest that survey methods may not be the most efficient for learning about anal cancer screening recency in this population. First, previous studies have suggested that ever being screened for anal cancer is low, hovering between 10% and 14% (15, 18, 19). While our study suggests that the recency of screening increased compared to other studies, we argue that more targeted methods to reach these populations would be more effective. This includes using electronic health record data, partnering with local HIV clinics, and engaging community members through community-engaged research. Second, MSM undergo several exams and tests involving the anus, including colonoscopies, prostate exams, and routine sexually transmitted infection (STI) checks. Increasing transparency and emphasizing differences between exams may facilitate better reporting of anal cancer screening experiences. To do so, self-reported anal cancer screenings should be validated against electronic health records. Validation is an important step to ensure the accuracy of self-reports for future research in settings where access to electronic health records is not available.

This study has limitations. This cross-sectional study recruited a small number of MSM; the majority were white, educated, young, and sexually active. The median age of our sample was 45 years, representing a younger group of men, a majority of whom just became eligible for screening under current guidelines. Due to our recruitment strategy, we sampled a few older men who would be at high risk for anal cancer and are critical to engage in consistent screening programs. As such, our findings may not be generalizable to other populations of MSM in Minnesota or other communities. Additionally, we only recruited participants who self-reported at least one experience with anal cancer screening and, therefore, are unable to comment on screening uptake. Furthermore, our study was underpowered to detect differences between MSM living with and without HIV. As such, larger studies are needed to confirm our findings. Lastly, given the number of MSM who initially reported anal cancer screening and later reported no history after reviewing descriptions (n = 14), it appears that anal cancer screening is often confused with other tests, leading to misclassification of our anal cancer screening outcomes. While it is unclear to what extent this biases our results, it is possible that participants over- or under-reported their experiences with screening. As such, we suggest that future research utilize electronic health records to verify screening history and results.

Despite these limitations, our study is one of the first to report the recency of anal cancer screening and follow-up care steps after guideline publication. While we did not observe statistically significant differences in recency of screening, methods used for screening, or variations in reported follow-up steps, we did note several important findings. Compared to previous literature, more MSM living with and without HIV self-reported being screened for anal cancer within the last year. While there were no statistically significant differences, future research is warranted to continue examining the recency of screening and factors associated with screening utilization. MSM living with HIV more frequently reported a history of infection with HPV or a related disease, and a greater proportion reported an abnormal screening result compared to HIV-negative MSM. In accordance with current guidelines, we suggest that providers should prioritize anal cancer screening for people living with HIV, especially in areas with few HRA providers. Additionally, we report that recommending providers varied by HIV status, providing opportunities to train providers on empathetic and transparent care to promote consistent screening.

## Data Availability

The original contributions presented in the study are included in the article/**Supplementary Material**. Further inquiries can be directed to the corresponding author.
