# Supplementary material for: Self-reported anal cancer screening experiences in MSM: recency, follow-up, and methodological insights
Source: Front Oncol. 2026 Apr 15;16:1689760. doi: 10.3389/fonc.2026.1689760 (PMC13124529; doi:10.3389/fonc.2026.1689760)
Supplement: Supplementary file 1 [file DataSheet1.pdf]

Q1 **In which state do you currently live?**

Drop down (Alabama-Wyoming or None of the above)

Page Break

JS *

Q2 **What is your current age in years?**

____

Page Break

JS

Q3 **What sex were you assigned at birth?** Meaning, what is the sex that appears on your original birth certificate.

- Male (M) (1)
- Female (F) (2)

Page Break

JS

Q4 **Have you ever had any type of sex with a man?**

- Yes (1)
- No (2)

Page Break

JS

Q5 **What was the result of your most recent test for HIV?**

○ HIV-positive (1)

○ HIV-negative (2)

○ Inconclusive or unknown (3)

○ Never been tested for HIV (4)

JS

Q6 **Have you ever been screened for anal cancer?** It can be hard to remember if you've been screened for anal cancer. If you are not sure, here are some descriptions.
Anal cancer screening is **NOT**: **X** done during a colonoscopy. **X** the same as prostate cancer screening. **X** the same tests you might get at a routine PrEP visit.
Anal cancer screening **IS**: + an anal Pap or HPV test, like a soft cotton swab that was inserted into the anus to collect cells. + a digital anorectal exam where your doctor used a lubricated, gloved finger (sometimes with numbing gel) to check for lumps in the anus. + high-resolution anoscopy where a small plastic tube called an anoscope was inserted while you were awake, without needing special bowel prep.

○ Yes (1)

○ No (2)

○ Not sure/don't remember (3)

Page Break

JS

Q7 **Are you a robot?**

End of Block: Eligibility

Start of Block: Consent

JS

CONSENT

Q8 Would you like to continue with the study?

- ◯ ***Yes***, I would like to continue. (1)
- ◯ ***No***, I would **not** like to continue. (2)

End of Block: Consent

Start of Block: Demographics

JS

The purpose of this section is to gather some information about you.

Page Break

Q9 How would you describe your gender identity today?

- ○ Cisgender man (1)
- ○ Transgender man (2)
- ○ Cisgender woman (3)
- ○ Transgender woman (4)
- ○ Non-binary (5)
- ○ Gender non-conforming or gender-queer (6)
- ○ Agender (7)
- ○ Two-Spirit (8)
- ○ Self-described (please specify): (9) ____
- ○ Prefer not to answer (10)

Page Break

Q10 How would you describe your sexual orientation today?

- Gay (1)
- Lesbian (2)
- Bisexual (3)
- Pansexual (4)
- Queer (5)
- Straight (6)
- Self-described (please specify): (7) ____
- Prefer not to answer (8)

Page Break

Q11 What is your current relationship status?

- Single (1)
- Partnered, in a monogamous relationship (2)
- Partnered, in an open relationship (3)
- Separated or divorced (4)
- Widowed (5)
- Self-described (please specify): (6) ____
- Prefer not to answer (7)

Page Break

Q12 What is the highest degree or level of school you have completed?

- ○ Less than high school (1)
- ○ High school diploma or equivalent (GED) (2)
- ○ Some college but no degree (3)
- ○ Technical degree (4)
- ○ Associate's degree (5)
- ○ Bachelor's degree (6)
- ○ Graduate or professional degree (7)
- ○ Prefer not to answer (8)

Page Break

Q13 The race and ethnicity categories listed are sociopolitical constructs and are not an attempt to define race and ethnicity biologically or genetically.   Which of the following best describe your race and ethnicity? *(check all that apply)*

- ▢ American Indian or Alaska Native (1)
- ▢ Asian (2)
- ▢ Black or African American (3)
- ▢ Native Hawaiian or Pacific Islander (4)
- ▢ White (5)
- ▢ Hispanic, Latino, or Spanish (6)
- ▢ Middle Eastern or North African (7)
- ▢ Self-described (please specify): (8) ____
- ▢ ⊗Prefer not to answer (9)

Page Break

Q14 Have you smoked at least 100 cigarettes or 5 packs in your lifetime?   ***Do not include:*** *electronic cigarettes (ecigarettes, njoy, bluetip, JUUL), herbal cigarettes, cigars, cigarillos, little cigars, pipes, bidis, kreteks, water pipes (hookahs) or marijuana.*

- ○ Yes (1)
- ○ No (2)
- ○ Not sure/don't know (3)
- ○ Prefer not to answer (4)

Page Break

Display this question:

If Have you smoked at least 100 cigarettes or 5 packs in your lifetime? Do not include: electronic c... = Yes

Q15 Do you now smoke cigarettes every day, some days, or not at all?

- Every day (1)
- Some days (2)
- Not at all (3)
- Not sure/don’t remember (4)
- Prefer not to answer (5)

Page Break

Q16 What is the current primary source of your health insurance?

- A plan purchased through an employer or union (including plans purchased through another person's employer) (1)
- A private non-governmental plan that you or another family member buys on your own (2)
- Medicare (3)
- Medicaid (4)
- Military related health care: TRICARE(CHAMPUS) / VA health care / CHAMP-VA (5)
- Indian Health Service (6)
- State sponsored health plan (7)
- Other governmental program (8)
- No coverage of any type (9)
- Not sure/don’t remember (10)
- Prefer not to answer (11)

Page Break

Q17 In what year were you born?
Drop down (1920-2024 or prefer not to answer)

Page Break

Q18 In which Minnesota county do you currently live?
Drop down (Aitkin-Yellow Medicine or prefer not to answer)

End of Block: Demographics

Start of Block: Sexual Behavior & Health

JS

Next, we're going to ask questions about your sexual behaviors and sexual health. **All data is confidential.**

Page Break

Q19 At what age did you have your first sexual intercourse? This includes oral, vaginal, or anal sex. ***Do not include:*** *masturbation, mutual masturbation, or genital contact that did not lead to intercourse as defined above.*

- ◯ Age: (1) ____
- ◯ Prefer not to answer (2)

Page Break

Q20 Please note there are *two questions* on this page.
Altogether in your life so far, how many different partners have you had anal sex with?

○ 0 partners (1)

○ 1 partner (2)

○ 2-5 partners (3)

○ 6-10 partners (4)

○ 11-25 partners (5)

○ 26-50 partners (6)

○ 51-100 partners (7)

○ More than 100 partners (8)

○ Prefer not to answer (9)

Q21 Which of these best describes how you worked out the answer above?

○ I just knew the number (1)

○ I remembered each partner and counted them up (2)

○ I estimated or guessed the number (3)

○ I remembered some partners and then added on an estimated number for others (4)

○ Some other way (please describe): (5)
____

○ Prefer not to answer (6)

Page Break

Q22 **In the last year**, how many different partners have you had receptive anal sex with (where you were the bottom)?

- 0 partners (1)
- 1 partner (2)
- 2-5 partners (3)
- 6-10 partners (4)
- 11-25 partners (5)
- 26-50 partners (6)
- 51-100 partners (7)
- More than 100 partners (8)
- Prefer not to answer (9)

Page Break

Display this question:

If In the last year, how many different partners have you had receptive anal sex with (where you wer... != 0 partners

Or In the last year, how many different partners have you had receptive anal sex with (where you wer... = Prefer not to answer

Q23 **In the last year**, how often did you use a condom when you had receptive anal sex (where you were the bottom)?

- ◯ Always (1)
- ◯ Sometimes (2)
- ◯ Never (3)
- ◯ Prefer not to answer (4)

Page Break

Q24 Have you ever been diagnosed with HIV or AIDS?

- ◯ Yes (1)
- ◯ No (2)
- ◯ Not sure/don't remember (3)
- ◯ Prefer not to answer (4)

Page Break

Display this question:

If Have you ever been diagnosed with HIV or AIDS? = Yes

Q25 At what age were you diagnosed with HIV?

- ◯ Age: (1) ____
- ◯ Prefer not to answer (2)

Page Break

Display this question:

If Have you ever been diagnosed with HIV or AIDS? = Yes

Q26 Are you currently taking antiretroviral therapy (ART) or highly active antiretroviral therapy (HAART)?

◯ Yes (1)

◯ No (2)

◯ Prefer not to answer (3)

Page Break

Display this question:

If Are you currently taking antiretroviral therapy (ART) or highly active antiretroviral therapy (HA... = Yes

Q27 At what age were you first prescribed ART or HAART?

◯ Age: (1) ____

◯ Prefer not to answer (2)

Page Break

Display this question:

If Have you ever been diagnosed with HIV or AIDS? = No

Or Have you ever been diagnosed with HIV or AIDS? = Not sure/don't remember

Q28 Have you ever heard of pre-exposure prophylaxis (PrEP)? Some brand names include Truvada, Descovy, and Appretude (Carbotegravir).

- Yes (1)
- No (2)
- Not sure/don't remember (3)
- Prefer not to answer (4)

Page Break

Display this question:

If Have you ever heard of pre-exposure prophylaxis (PrEP)? Some brand names include Truvada, Descovy... = Yes

Q29 Are you currently taking pre-exposure prophylaxis (PrEP)?

- Yes (1)
- No (2)
- Prefer not to answer (3)

Page Break

Q30 Have you ever been diagnosed with human papillomavirus (HPV) or an HPV-related disease?   This includes anal warts, oral warts, anal dysplasia/anal pre-cancer, or anal cancer.

- Yes (1)
- No (2)
- Not sure/don't remember (3)
- Prefer not to answer (4)

Page Break

Q31 Have you ever been diagnosed with a sexually transmitted infection (STI)?

- Yes (1)
- No (2)
- Not sure/don't remember (3)
- Prefer not to answer (4)

Page Break

Display this question:

If Have you ever been diagnosed with a sexually transmitted infection (STI)? = Yes

Or Have you ever been diagnosed with a sexually transmitted infection (STI)? = Not sure/don't remember

Q32 Which of the following sexually transmitted infections (STIs) have you ever been diagnosed with? *(check all that apply)*

▢ Chlamydia (1)

▢ Gonorrhea (2)

▢ Syphilis (3)

▢ Herpes simplex virus (HSV) (4)

▢ Hepatitis A (5)

▢ Hepatitis B (6)

▢ Hepatitis C (7)

▢ Trichomoniasis (Trich) (9)

▢ Non-specific urethritis (NSU) or non-gonococcal urethritis (NGU) (10)

▢ Pubic lice/crabs (11)

▢ Monkeypox/Mpox (12)

▢ Something else (please specify): (13) ____

▢ ⊗Never been diagnosed with a STI (15)

▢ ⊗Prefer not to answer (14)

End of Block: Sexual Behavior & Health

Start of Block: HPV Vaccination

JS

You are half way through the survey! Keep going! This section will ask if you remember receiving certain vaccines.

Page Break

Q33 Have you ever received any doses of the HPV vaccine? The human papillomavirus (HPV) vaccine is given to prevent HPV infection and genital warts. The brand name for the vaccine is Gardasil.

- Yes (1)
- No (2)
- Not sure/don't remember (3)
- Prefer not to answer (4)

Skip To: End of Block If Have you ever received any doses of the HPV vaccine? The human papillomavirus (HPV) vaccine is gi... = Prefer not to answer

Skip To: End of Block If Have you ever received any doses of the HPV vaccine? The human papillomavirus (HPV) vaccine is gi... = Not sure/don't remember

Page Break

Display this question:

If Have you ever received any doses of the HPV vaccine? The human papillomavirus (HPV) vaccine is gi... = No

Q34 Were you ever offered the HPV vaccine?

- Yes, I was offered but refused it (1)
- No, I was never offered it (2)
- Prefer not to answer (3)

Skip To: End of Block If Were you ever offered the HPV vaccine? = No, I was never offered it

Skip To: End of Block If Were you ever offered the HPV vaccine? = Prefer not to answer

Skip To: End of Block If Were you ever offered the HPV vaccine? = Yes, I was offered but refused it

Page Break

Display this question:

If Have you ever received any doses of the HPV vaccine? The human papillomavirus (HPV) vaccine is gi... = Yes

Q35 How many doses of the HPV vaccine have you ever received? Please select your best guess if you do not remember.

- 1 (1)
- 2 (2)
- 3 or more (3)
- Not sure/don't remember (4)
- Prefer not to answer (5)

Page Break

Display this question:

If How many doses of the HPV vaccine have you ever received? Please select your best guess if you do... != Prefer not to answer

Q36 How old were you when you received your first dose of the HPV vaccine? You can write in your best guess if you do not remember.

- Age: (1) ____
- Prefer not to answer (2)

End of Block: HPV Vaccination

Start of Block: Anal Cancer

JS

Just a few questions left! Now, we're going to ask about your experiences with anal cancer screenings.   Please remember the following ***before*** answering the next questions:
An anal Pap test is used to detect cancer cells or anal HPV. It is not a test for chlamydia or gonorrhea, like you may get at a routine PrEP visit.
Anal cancer screening is not conducted during a routine colonoscopy.
Prostate cancer screening is **not** the same as screening for anal cancer.

Page Break

Q37 Have you ever been screened for anal cancer?

◯ Yes (1)

◯ No (2)

◯ Prefer not to answer (3)

Skip To: Q47 If Have you ever been screened for anal cancer? = Prefer not to answer

Skip To: Q47 If Have you ever been screened for anal cancer? = No

Page Break

Q38 Who first recommended anal cancer screening to you?

- ○ Infectious disease or HIV provider (1)
- ○ Primary care provider (2)
- ○ Friend (6)
- ○ Partner (7)
- ○ Family member (8)
- ○ Not sure/don't remember (3)
- ○ Someone else (please specify): (4) ____
- ○ Prefer not to answer (5)

Page Break

Q39 What type of healthcare provider first performed your anal cancer screening?

- ○ Infectious disease or HIV provider (1)
- ○ Primary care provider (2)
- ○ Not sure/don't remember (3)
- ○ Some other provider (please specify): (4) ____
- ○ Prefer not to answer (5)

Page Break

Q40 At what age did you start getting screened for anal cancer?

- ◯ Age: (1) ____

- ◯ Prefer not to answer (2)

Page Break

Display this question:

If Have you ever been diagnosed with HIV or AIDS? = Yes

Q41 Did you **start** anal cancer screening *before* or *after* you were diagnosed with HIV/AIDS?

- ◯ I started screening for anal cancer ***before*** my HIV diagnosis (1)

- ◯ I started screening for anal cancer ***after*** my HIV diagnosis (2)

- ◯ Not sure/don't remember (3)

- ◯ Prefer not to answer (4)

Page Break

Q42 Have you ever been screened for anal cancer using any of these methods? *(check all that apply)*

▢ **An anal Pap test** (moistened cotton swab is inserted into the anal canal to collect cells for testing) (1)

▢ **An anal HPV test** (a moistened cotton swab is inserted into the anal canal) (2)

▢ **A digital anal exam** (your healthcare provider would insert a lubricated finger with a local anesthetic into the anus to feel for lumps, ulcerations, and/or masses) (3)

▢ **A procedure called high-resolution anoscopy or HRA** (no bowel prep, you were likely awake, performed with a small plastic scope called an anoscope) (4)

▢ Another method (please specify): (5) ____

▢ ⊗Not sure/don't remember (6)

▢ ⊗Prefer not to answer (7)

Skip To: Q44 If Have you ever been screened for anal cancer using any of these methods? (check all that apply) = Not sure/don't remember

Skip To: Q44 If Have you ever been screened for anal cancer using any of these methods? (check all that apply) = Prefer not to answer

Page Break

Display this question:

If Have you ever been screened for anal cancer using any of these methods? (check all that apply) != Not sure/don't remember

Or Have you ever been screened for anal cancer using any of these methods? (check all that apply) != Prefer not to answer

JS

Q43 When were you last screened for anal cancer using each method?

Display this choice:

If Have you ever been screened for anal cancer using any of these methods? (check all that apply) = An anal Pap test (moistened cotton swab is inserted into the anal canal to collect cells for testing)

Display this choice:

If Have you ever been screened for anal cancer using any of these methods? (check all that apply) = An anal HPV test (a moistened cotton swab is inserted into the anal canal)

Display this choice:

If Have you ever been screened for anal cancer using any of these methods? (check all that apply) = A digital anal exam (your healthcare provider would insert a lubricated finger with a local anesthetic into the anus to feel for lumps, ulcerations, and/or masses)

Display this choice:

If Have you ever been screened for anal cancer using any of these methods? (check all that apply) = A procedure called high-resolution anoscopy or HRA (no bowel prep, you were likely awake, performed with a small plastic scope called an anoscope)

Display this choice:

If Have you ever been screened for anal cancer using any of these methods? Text Response Is Not Empty

	Less than 1 year ago (6)	1 to 3 years ago (2)	More than 3 years ago (3)	Not sure/don't remember (4)	Prefer not to answer (5)
Display this choice: If Have you ever been screened for anal cancer using any of these methods? (check all that apply) = An anal Pap test (moistened cotton swab is inserted into the anal canal to collect cells for testing) An anal Pap test (1)	○	○	○	○	○
Display this choice: If Have you ever been screened for anal cancer using	○	○	○	○	○

any of these methods? (check all that apply) = An anal HPV test (a moistened cotton swab is inserted into the anal canal) An anal HPV test (2)					
Display this choice: If Have you ever been screened for anal cancer using any of these methods? (check all that apply) = A digital anal exam (your healthcare provider would insert a lubricated finger with a local anesthetic into the anus to feel for lumps, ulcerations, and/or masses) A digital anal exam (3)	○	○	○	○	○
Display this choice: If Have you ever been screened for anal cancer using any of these methods? (check all that apply) = A procedure called high-resolution anoscopy or HRA (no bowel prep, you were likely awake, performed with a small plastic scope called an anoscope) High-resolution anoscopy (HRA) (4)	○	○	○	○	○
Display this choice: If If Have you ever been screened for anal cancer using any of these methods? Text Response Is Not Empty ${Q42/ChoiceTextEntryValue/5} (5)	○	○	○	○	○

Page Break

Q44 **In your lifetime**, approximately how many times in total have you been screened for anal cancer?

○ Total number of screenings: (1) ____

○ Prefer not to answer (2)

Page Break

Q45 Has a healthcare provider ever told you that you had an abnormal anal cancer screening result?

○ Yes (1)

○ No (2)

○ Not sure/don't remember (3)

○ Prefer not to answer (4)

Page Break

Display this question:

If Has a healthcare provider ever told you that you had an abnormal anal cancer screening result? = Yes

Q46 Based on your abnormal anal cancer screening result, did your healthcare provider recommend any of the following? *(check all that apply)*

- ▢ Re-screening or collecting the test again as soon as possible (1)
- ▢ Screening again in a year (2)
- ▢ High-resolution anoscopy (HRA) for diagnosis and/or treatment (3)
- ▢ ⊗No follow-up (4)
- ▢ ⊗Prefer not to answer (5)

Page Break

Q47 Has a healthcare provider ever told you that you have anal cancer?

- ○ Yes (1)
- ○ No (2)
- ○ Prefer not to answer (3)

Page Break

Display this question:

If Has a healthcare provider ever told you that you have anal cancer? = Yes

Q48 At what age were you first diagnosed with anal cancer?

- ○ Age: (1) ____
- ○ Prefer not to answer (2)

Page Break

Display this question:

If Has a healthcare provider ever told you that you have anal cancer? = Yes

Q49 At which stage were you diagnosed with anal cancer?

- Stage 1 (1)
- Stage 2 (2)
- Stage 3 (3)
- Stage 4 (4)
- Not sure/don't remember (5)
- Prefer not to answer (6)

End of Block: Anal Cancer

Start of Block: Payment

JS

ou completed the survey! Our study team values your time and effort. Please continue to the next page for gift card options.

Page Break

Q50 As a thank you, would you like to receive a $15 Amazon e-gift card? *Note: it may take up to 3 weeks to receive your gift card.*

- Yes (1)
- No (2)

Skip To: If As a thank you, would you like to receive a $15 Amazon e-gift card? Note: it may take up to 3 wee... = No

Page Break

Display this question:

If As a thank you, would you like to receive a $15 Amazon e-gift card? Note: it may take up to 3 wee... = Yes

*

Q51 Please enter your email address

____

Display this question:

If As a thank you, would you like to receive a $15 Amazon e-gift card? Note: it may take up to 3 wee... = Yes

*

Q52 Please re-enter your email address. We recommend taking a screenshot of this page with your email address entered for your records.

____

Page Break

Q53 You have the option to provide some additional information about yourself to receive an **extra $5 gift card**. Completing this research activity is *optional* and will take about 2 minutes. The form for this research activity will open in a new window. You may choose not to participate in this research activity and still finish the study by clicking the arrow at the bottom of the screen.
The Minnesota Department of Health will *not* see any of the information you provided up to this point.
Before clicking the button below, please copy this five digit survey ID and be ready to paste it in the form: **${e://Field/RandomID}**

JS

Please click **"Submit"** to finish your survey.
