# Supplementary material for: Self-reported anal cancer screening experiences in MSM: recency, follow-up, and methodological insights
Source: Front Oncol. 2026 Apr 15;16:1689760. doi: 10.3389/fonc.2026.1689760 (PMC13124529; doi:10.3389/fonc.2026.1689760)
Supplement: Supplementary file 2 [file Table1.docx]

Supplementary Material

# Supplementary Table

| Supplementary Table 1. Comparison of demographics for participants with and without missing anal cancer outcomes (n=52). | | | | | |
| --- | --- | --- | --- | --- | --- |
|  | **Missing (n=14)** | | **Non-missing (n=38)** | | **p-value** |
|  | **N** | **%** | **N** | **%** |  |
| **Current age (Median, IQR)** | | | | | 0.24 |
|  | 43 (26-47) | | 46 (40-57) | |  |
| **Race** | | | | | |
| American Indian or Alaska Native | 0 | 0.0% | 1 | 2.6% | 0.12 |
| Asian | 2 | 14.3% | 1 | 2.6% |  |
| Black or African American | 2 | 14.3% | 4 | 10.5% |  |
| Hispanic/Latino | 2 | 14.3% | 1 | 2.6% |  |
| More than one | 2 | 14.3% | 3 | 7.9% |  |
| White | 6 | 46.2% | 28 | 73.7% |  |
| **Sexual orientation** | | | | | |
| Gay | 12 | 85.7% | 34 | 89.5% | 0.69 |
| Bisexual | 1 | 7.1% | 1 | 2.6% |  |
| Pansexual | 0 | 0.0% | 1 | 2.6% |  |
| Queer | 1 | 7.1% | 2 | 5.3% |  |
| **Gender identity** | | | | | |
| Cisgender man | 12 | 85.7% | 33 | 89.2% | >0.99 |
| Non-Binary | 0 | 0.0% | 1 | 2.7% |  |
| Two-Spirit | 0 | 0.0% | 1 | 2.7% |  |
| Other identity | 1 | 7.1% | 2 | 5.4% |  |
| **Education** | | | | | |
| High School or GED | 3 | 21.4% | 3 | 7.9% | 0.37 |
| Some college | 0 | 0.0% | 5 | 13.2% |  |
| Technical degree | 0 | 0.0% | 1 | 2.6% |  |
| Associate's degree | 1 | 7.1% | 2 | 5.3% |  |
| Bachelor's degree | 7 | 50.0% | 13 | 34.2% |  |
| Graduate or professional degree | 3 | 21.4% | 14 | 36.8% |  |
| **History of smoking** | | | | | |
| Yes | 5 | 35.7% | 17 | 44.7% | 0.75 |
| No | 9 | 64.3% | 21 | 55.3% |  |
| **HIV status** | | | | | |
| HIV-positive | 2 | 14.3% | 14 | 36.8% | 0.18 |
| HIV-negative | 12 | 85.7% | 24 | 63.2% |  |
| **History of STI (excluding HPV and HIV)** | | | | | |
| Yes | 11 | 76.9% | 33 | 86.8% | 0.37 |
| No | 3 | 23.1% | 5 | 13.2% |  |
| **History of HPV infection or related disease** | | | | | |
| Yes | 2 | 14.3% | 15 | 39.5% | 0.05* |
| No | 12 | 85.7% | 22 | 57.9% |  |
| Not sure/don't remember | 0 | 0.0% | 1 | 2.6% |  |
| **Health insurance** | | | | | |
| Insured | 12 | 85.7% | 38 | 100.0% | 0.07 |
| Uninsured | 2 | 14.3% | 0 | 0.0% |  |
| **Self-reported HPV vaccination status** | | | | | |
| Vaccinated | 7 | 50.0% | 18 | 47.4% | 0.71 |
| Unvaccinated | 7 | 50.0% | 16 | 42.1% |  |
| Unsure | 0 | 0.0% | 4 | 10.5% |  |
| **Self-reported number of doses (only vaccinated)** | | | | | |
| 1 | 1 | 14.3% | 1 | 5.6% | >0.99 |
| 2 | 2 | 28.6% | 5 | 27.8% |  |
| 3+ | 4 | 57.1% | 10 | 55.6% |  |
| Not sure/don't remember | 0 | 0.0% | 2 | 11.1% |  |
| **Self-reported age at first HPV dose (Median, IQR)** | | | | | |
|  | 21 (18-34) | | 32 (30-40) | | 0.06 |
| **Age at sexual debut (Median, IQR)** | | | | | |
|  | 18 (15-23) | | 16 (14-19) | | >0.99 |
| **Lifetime number of anal sex partners** | | | | | |
| 2-5 | 2 | 14.3% | 1 | 2.6% | 0.03* |
| 6-10 | 0 | 0.0% | 0 | 0.0% |  |
| 11-25 | 1 | 7.1% | 5 | 13.2% |  |
| 26-50 | 2 | 14.3% | 3 | 7.9% |  |
| 51-100 | 5 | 35.7% | 4 | 10.5% |  |
| >100 | 4 | 28.6% | 25 | 65.8% |  |
| **Number of RAI partners in last year** | | | | | |
| 0 | 1 | 7.1% | 9 | 23.7% | 0.18 |
| 1 | 2 | 14.3% | 3 | 7.9% |  |
| 2-5 | 4 | 28.6% | 10 | 26.3% |  |
| 6-10 | 3 | 21.4% | 4 | 10.5% |  |
| 11-25 | 1 | 7.1% | 7 | 18.4% |  |
| 26-50 | 1 | 7.1% | 5 | 13.2% |  |
| 51-100 | 0 | 0.0% | 0 | 0.0% |  |
| >100 | 2 | 14.3% | 0 | 0.0% |  |
| **Condom use in last year (only those with ≥1 RAI partner in past year)** | | | | | |
| Always | 2 | 15.4% | 0 | 0.0% | 0.08 |
| Sometimes | 6 | 46.2% | 11 | 37.9% |  |
| Never | 5 | 38.5% | 18 | 62.1% |  |
| IQR: interquartile range; GED, General Educational Development; HIV: human immunodeficiency virus; STI: sexually transmitted infection; HPV: human papillomavirus; RAI: receptive anal intercourse  * indicates p-value <0.05 | | | | | |
